# Human Menstrual Blood-Derived Stem Cells Inhibit the Proliferation of HeLa Cells via TGF-*β*1-Mediated JNK/P21 Signaling Pathways

**DOI:** 10.1155/2019/9280298

**Published:** 2019-05-19

**Authors:** Qian-Yu Liu, Feng Ruan, Jing-Yuan Li, Li Wei, Ping Hu, Hou-Wen Chen, Quan-Wen Liu

**Affiliations:** ^1^The National Engineering Research Center for Bioengineering Drugs and the Technologies, Institute of Translational Medicine, Nanchang University, Nanchang 330031, China; ^2^Department of Emergency Medicine, Second Affiliated Hospital Zhejiang University School of Medicine, Hangzhou 310009, China; ^3^School of Life and Science, Nanchang University, Nanchang 330031, China; ^4^State Key Laboratory for Nuclear Resources and Environment, and School of Biology, Chemistry and Material Science, East China University of Technology, Nanchang, Jiangxi 34000, China

## Abstract

Human menstrual blood-derived stem cells (hMBSCs) are a novel type of mesenchymal stem cells (MSCs) that have a high proliferative rate, multilineage differentiation potential, low immunogenicity, and low oncogenicity, making them suitable candidates for regenerative medicine. The therapeutic efficacy of hMBSCs has been demonstrated in some diseases; however, their effects on cervical cancer remain unclear. In the present study, we investigated whether hMBSCs have anticancer properties on cervical cancer cells in vivo and in vitro, which has not yet been reported. In vitro, transwell coculturing experiments revealed that hMBSCs suppress the proliferation and invasion of HeLa cervical cancer cells by inducing G0/G1 cell cycle arrest. In vivo, we established a xenografted BALB/c nude mouse model by subcutaneously coinjecting HeLa cells with hMBSCs for 21 days. We found that hMBSCs significantly decrease the average volume and average weight of xenografted tumors. ELISA, TGF-*β*1 antibody, and recombinant human TGF-*β*1 (rhTGF-*β*1) were used to analyze whether TGF-*β*1 contributed to cell cycle arrest. We found that hMBSC-secreted TGF-*β*1 and rhTGF-*β*1 induced cell cycle arrest and increased the expression of phospho-JNK and phospho-P21 in HeLa cells, which was mostly reversed by TGF-*β*1 antibody. These results indicate that hMBSCs have antitumor properties on cervical cancer in vitro and in vivo, mediated by the TGF-*β*1/JNK/p21 signaling pathway. In conclusion, this study suggests that hMBSC-based therapy is promising for the treatment of cervical cancer.

## 1. Introduction

Cervical cancer is a common malignancy and has been ranked as the second leading cause of cancer-related deaths in women [1], with about 52,900 new cases and 275,000 deaths every year [2, 3]. Despite the improvement in preventative, diagnostic, and therapeutic strategies, the five-year survival rate for patients with advanced stages remains as low as 40%, resulting in a large number of cancer-related deaths [4, 5]. Thus, novel strategies for the treatment of cervical cancer are greatly needed. Currently, stem cells are being explored as a promising candidate for cancer therapy.

Human mesenchymal stem cells (MSCs), a cell population with low immunogenicity and low oncogenicity [6], are multipotent cells with the ability to self-renew and differentiate into adipocyte, osteoblast, and chondrocyte lineages [7–9]. A series of studies have shown that MSCs play a critical role in regulating tumor initiation and progression by affecting the invasion, migration, or apoptosis resistance of tumor cells; however, the effects remain controversial. Tang et al. demonstrated that MSCs enhanced the growth of hepatocellular carcinoma [10]. Ding et al. found that ovarian mesenchymal stem cells promote proliferation, sphere and colony formation, and tumorigenesis of ovarian cancer cells [11]. Several other studies have shown that MSCs have tumorigenic effects in a variety of cancer cells in vitro and are recruited to tumor sites and contribute to tumor growth and progression in vivo, such as in breast cancer [12, 13], prostate cancer [14], hepatocellular carcinoma [15, 16], head and neck cancer [17], and gastric cancer [18]. In contrast, several reports have indicated that hMSCs inhibit tumor growth and can be used for effective cytotherapy in diverse tumor models [19, 20]. For instance, Ho et al. demonstrated that bone marrow-derived MSCs can be specifically recruited to tumor sites and reduce the tumor volume of glioma [21]. Yulyana et al. showed that in a model of liver cancer, condition media derived from human fetal MSCs can inhibit hepatoma carcinoma cell proliferation and reduce tumor burden [22, 23]. Ma et al. found that human umbilical cord mesenchymal stem cells significantly inhibited the growth of breast cancer cells in vitro and in vivo, likely in a cell cycle arrest-related manner [24]. Additionally, amniotic fluid and amniotic membrane-derived MSCs can induce cell cycle arrest in the G0/G1 phase and significantly reduce the proliferation of diverse cancer cell lines, including HeLa cells (human cervical epithelioid carcinoma cell line), Saos cells (human osteosarcoma cell line) [25, 26], Skov-3 (human epithelial ovarian cancer cell line) [27], KG1 cells (human acute myelogenous leukemia cell line), Jurkat cells (human T-cell leukemia cell line), and U937 cells (human monocytic cell line obtained from histiocytic lymphoma). Although studies on the anticancer effects of MSCs from different sources have been widely reported, few have focused on human menstrual blood-derived stem cells (hMBSCs). hMBSCs have phenotypes and properties similar to bone marrow (BM) MSCs, including high proliferative capabilities, multilineage differentiation potential, and surface marker expression [28, 29]. hMBSCs possess low immunogenicity, low oncogenicity, and remarkable regenerative capacity [30, 31]. Furthermore, the isolation procedure of hMBSCs is safe, simple, and without ethical issues [32]. A large number of reports have demonstrated the therapeutic potential of hMBSCs in different diseases, such as Alzheimer's disease [29], acute lung injury [33], cardiac fibrosis [28, 34], fulminant hepatic failure [35], and diabetes [36]. However, the therapeutic potential of hMBSCs in cancer treatment, including cervical cancer, has not yet been reported.

Our group recently isolated hMBSCs from women's menstrual blood and characterized their morphology, phenotypic profiles, pluripotency, and growth potency. To determine whether hMBSCs have antitumor effects on HeLa cells in vitro, hMBSC-conditioned medium (CM) and a transwell coculture system were used to detect the influences of hMBSC-secreted factors on the proliferation, invasion, and cell cycle progression of HeLa cells. In vivo, we established HeLa/NIH 3T3- and HeLa/hMBSC-coinjected xenografted BALB/c nude mouse models. We found that hMBSCs had anticancer effects when cocultured or coinjected with HeLa cells by inducing cell cycle arrest. ELISA, TGF-*β*1 antibody, and recombinant human TGF-*β*1 (rhTGF-*β*1) were used to confirm that TGF-*β*1 was the cell cycle regulatory cytokine secreted from hMBSCs to exert an inhibitory effect on HeLa cells. We also found that TGF-*β*1 can upregulate the expression of phospho-JNK and phospho-P21 in HeLa cells. Taken together, the results demonstrate that TGF-*β*1 released by hMBSCs inhibit cervical cancer growth in vivo and in vitro by activating JNK/P21 signaling.

## 2. Materials and Methods

### 2.1. Isolation of hMBSCs and Production of CM

The hMBSCs were isolated from female donors as previously described [28, 36] with slight modifications. The isolation procedure and informed consent form signed by volunteer donors were both approved by the Ethics Committee of Nanchang University. Menstrual blood samples were collected with a DivaCup (Kitchener, ON, Canada) from healthy menstruating women. Mononuclear cells were separated by density gradient centrifugation with Ficoll-Paque (Thermo Fisher Scientific Life Sciences, Oakwood Village, OH). Briefly, 8 ml menstrual blood was slowly layered on top of 4 ml Ficoll-Paque premium solution in a 15 ml polypropylene centrifuge tube. The tube was put into a swinging bucket centrifuge at 1800 rpm for 20 min, dividing the sample into three layers: the upper layer contained plasma, the mononuclear cells were undisturbed in the interlayer, and the bottom layer mostly contained erythrocytes. We then carefully removed the upper layer. The interlayer cells were then collected and washed with PBS three times. The purified mononuclear cells were cultured in *α*-MEM medium (Thermo Fisher) containing 10% FBS, 1% glutamine, and 1% penicillin/streptomycin (Thermo Fisher), supplemented with 18% Chang B and 2% Chang C (Irvine Scientific) at 37°C in a 5% CO_2_ humidified atmosphere. The media were changed every 2-3 days until adherent cells grew to 80%–90% confluency, and then the cells were subcultured using 0.25% trypsin (Thermo Fisher). The cells during passages 2 to 5 were used for subsequent experiments.

hMBSC-CM was prepared as follows: 5 × 10^5^ hMBSCs were placed in a 10 cm dish (Corning, NY, USA) and cultured in a normal medium. Once the cells reached 80% confluency, the medium was changed to H-DMEM (Thermo Fisher) containing 100 U/ml penicillin/streptomycin. hMBSC-CM was collected 48 h later and centrifuged at 1000 rpm at room temperature for 5 min, the supernatant was collected and concentrated tenfold (10X) using an Amicon® Ultra 3K device (MilliporeSigma, USA).

### 2.2. Identification of hMBSCs by Flow Cytometry

Phenotypic analyses of cultured hMBSCs were performed using standard flow cytometry methods. Passage 3 hMBSCs were collected in fluorescence-activated cell sorting (FACS) tubes (BD Biosciences, Franklin Lakes, NJ) at a concentration of 1 × 10^6^ cells/ml in stain FACS buffer (PBS containing 2% FBS), and then stained with FITC-conjugated antibodies against human CD29, CD90, CD45, HLA-DR, CD80, and CD40, phycoerythrin- (PE-) conjugated antibodies against human CD73, CD105, CD34, HLA-ABC, and CD86, and their isotype controls (all from BD Biosciences) at 4°C for 30 min in the dark. After washing twice, the cells were resuspended in 200 *μ*l of PBS and acquired by a FACSCalibur instrument (BD Biosciences). Dates were analyzed using the FLOWJO™ software (TreeStar, Inc., Ashland, OR, USA).

### 2.3. Immunofluorescence

Immunofluorescence experiments were carried out following our previously reported protocols [37]. Briefly, cells growing on the glass slide were fixed with 4% paraformaldehyde for 15 min and permeabilized using 0.25% Triton X-100 diluted in PBS for 10 min at room temperature. To block unspecific epitopes, cells were incubated with PBS containing 1% BSA and 0.1% Tween-20 for 1 h. To evaluated the pluripotency of hMBSCs, we used the following antibodies: rabbit anti-OCT_4_ (5 *μ*g/ml, Abcam, Nanchang, China), mouse anti-SSEA-4 (15 *μ*g/ml, Abcam), and rabbit anti-Nanog (1 : 200, Abcam). The fixed cells were incubated overnight at 4°C with primary antibodies followed by incubation with secondary donkey anti-mouse or anti-rabbit antibodies conjugated to either Alexa Fluor 488 or Alexa Fluor 568 (Jackson, Nanchang, China). Nuclei were counterstained with DAPI (Thermo Fisher).

### 2.4. Adipogenic and Osteogenic Differentiation

Passage 3 hMBSCs were seeded at a density of 1.5 × 10^5^ cells/well in a six-well plate. When the cells reached 100% confluence, the OriCell™ human mesenchymal stem cell adipogenic differentiation medium (Cyagen Biosciences, Shanghai, China) was added to wells according to the manufacturer's instruction. After 28 days of induction, Oil red O (Cyagen Biosciences) staining was performed to assess the differentiation potential of adipogenesis formation of intracellular lipid droplets. For osteogenic differentiation, hMBSCs were cultured with OriCell™ human mesenchymal stem cell osteogenic differentiation medium (Cyagen Biosciences) for 10 days and 21 days to analyze the middle and late stages of osteogenic differentiation. The differentiation potential for osteogenesis was assessed through alkaline phosphatase (ALP) (Cyagen Biosciences) staining at the middle stage and Alizarin Red (pH 4.2, 40 mM) (Cyagen Biosciences) staining at the late stage.

### 2.5. In Vitro Coculture Experiment

The human cervical cancer cell line HeLa and fibroblast cell line NIH 3T3 were obtained from the American Type Culture Collection (ATCC, Manassas, VA) and cultured in high-glucose DMEM (Thermo Fisher) containing 10% FBS, 100 U/ml penicillin, and 100 *μ*g/ml streptomycin at 37°C in a humidified atmosphere with 5% CO_2_.

**Figure 1:**
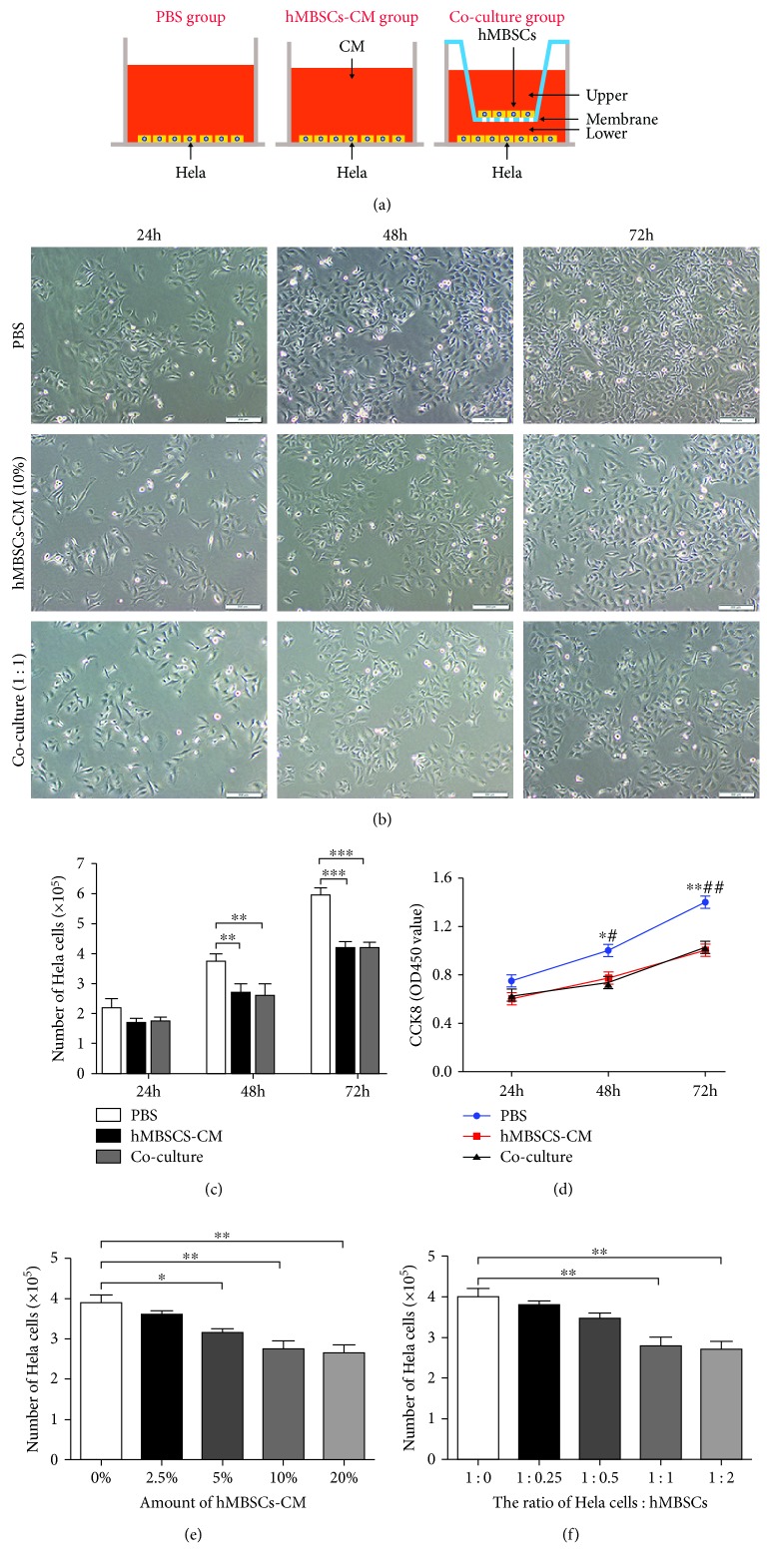
hMBSCs and hMBSC-CM inhibit the proliferation of HeLa cells in vitro in a paracrine manner. (a) Schematic diagram of the PBS control group, hMBSC-CM group, and hMBSC coculture group. (b) Representative images of the cell proliferation assay treated with PBS, hMBSC-CM (10%), and hMBSCs (1 : 1) at different time points. (c) The effects of PBS, hMBSC-CM, and hMBSC coculture on HeLa cell proliferation were tested by cell counting assay (*n* = 3). (d) Cell viability was measured 24 h, 48 h, and 72 h after treatment with PBS, hMBSC-CM, and hMBSC coculture using a CCK-8 assay. The results show that hMBSC-CM and hMBSC coculture enhances the inhibition of HeLa cell proliferation. (e) The number of HeLa cells was measured 48 h after treatment with different concentrations (2.5%, 5%, 10%, or 20%) of hMBSC-CM (10X). (f) Cell viability was measured 48 h after coculturing in the presence of different concentrations of hMBSCs (at a ratio of HeLa cells : hMBSCs of 4 : 1, 2 : 1, 1 : 1, or 1 : 2) (*n* = 3; ^∗^compared with the hMBSC-CM group; ^#^compared with the hMBSC coculture group).

HeLa cells were trypsinized and seeded in a 6-well dish at 1.5 × 10^5^ cells/well. For the PBS-treated control group, cells were incubated with 3 ml H-DMEM containing 10% FBS and 1% penicillin/streptomycin. For the hMBSC-CM group, HeLa cells were cultured with 3 ml H-DMEM supplemented with 10% hMBSC-CM (10X), 10% FBS, and 1% penicillin/streptomycin. For the coculture group, a coculture transwell chamber (2.4 cm diameter, 0.4 *μ*m pore size; Corning) was used to assess the effects of hMBSCs on HeLa cells in vitro. HeLa cells were seeded into the lower chamber at a concentration of 1.5 × 10^5^ cells/well in 2.0 ml of H-DMEM with 10% FBS, and 1.5 × 10^5^ hMBSCs were seeded in the upper compartment in 1.0 ml of the same medium (Figure 1(a)). Samples were collected after culturing for 24, 48, and 72 h.

### 2.6. Tumor Cell Proliferation, Apoptosis, and Cell Cycle Analysis

Cell proliferation was determined at indicated time points using the CCK-8 kit (Dojindo Laboratories, Kumamoto, Japan), following the manufacturer's protocol. We added 10% of CCK-8 solution to each well for 3 h before measuring the absorbance at 450 nm using a microplate spectrophotometer (Bio-Rad).

For the apoptosis assays, 1.0 × 10^5^ cells were collected from each sample and resuspended in 100 *μ*l Annexin V binding solution containing 5 *μ*l Annexin V-FITC and 5 *μ*l propidium iodide (PI) solution (Dojindo). After incubation for 15 min at room temperature, cells were washed in PBS, centrifuged at 100 rpm for 5 min, and resuspended in 400 *μ*l Annexin V Binding Buffer. For cell cycle assays, cells were fixed in 70% precooled ethanol on ice for 2 h, centrifuged at 100 rpm for 5 min, and resuspended with 400 *μ*l PI and 100 *μ*l RNaseA (Dojindo). After a 30 min incubation at 4°C, cells were washed and resuspended with PBS. Both the apoptosis assays and cell cycle assays were run and analyzed with BD Jazz.

### 2.7. Wound-Healing Assay

HeLa cells were seeded in a 6-well plate at a concentration of 1.5 × 10^5^ cells/well. When the cells reached 100% confluency, a sterile 200 *μ*l pipette tip was used to create a scratch wound on the cell monolayer. The cell debris was washed gently with PBS, and the cells were treated with PBS, hMBSC-CM, or hMBSCs. The dishes were incubated at 37°C in a 5% CO_2_ air atmosphere for 24 h and 48 h. Images were acquired at 24 h and 48 h time points and measured using the Image-Pro Plus 6.0 software.

### 2.8. Invasion Assays

BD Matrigel™ invasion chambers (8 *μ*m pore size, MA, USA) were used to investigate the effect of hMBSC-CM and hMBSCs on HeLa cell invasion. HeLa cells (1 × 10^5^) were added to the upper chamber, and hMBSCs or conditioned media derived from hMBSCs were added to the bottom well. Controls contained only DMEM with 10% FBS in the bottom well. After incubation for 48 h at 37°C, the chambers were fixed. Noninvaded cells were scraped off the upper side of the chamber, and the insert was stained with crystal violet. Images were taken using a phase-contrast microscope.

### 2.9. Western Blot Analysis

Protein extracts were prepared from HeLa cells with lysis buffer containing 25 mM Hepes (pH 7.4), 1% NP40, 137 mM NaCl, 10% glycerol, 50 mM NaF, and complete protease inhibitor cocktail (Roche, Mannheim, Germany). Cell extracts were centrifuged at 13,000 rpm at 4°C for 10 min to remove insoluble debris and chromosomal DNA. In total, 60 *μ*g of total cell protein was run on 10% denaturing SDS-PAGE gels, then transferred to nitrocellulose membranes (Bio-Rad), which were incubated with primary antibodies anti-GAPDH (1 : 1000, rabbit monoclonal, Santa Cruz), anti-Bax (1 : 1000, rabbit polyclonal, Cell Signaling Technology), anticaspase 3 (1 : 1000, rabbit polyclonal, Abcam), anti-PCNA (1 : 1000, mouse monoclonal, Abcam), and anti-BCL2 (1 : 1000, rabbit monoclonal, Abcam) at 4°C overnight. Blots were detected with horseradish peroxidase- (HRP-) conjugated goat anti-rabbit or rabbit anti-mouse secondary antibody (Invitrogen) for 1 h at room temperature. Images were quantified using the SuperSignal West Pico or Femto chemiluminescent detection system (Pierce).

### 2.10. HeLa Cell Xenograft Model and Whole-Body Fluorescent Imaging

Male BALB/c nude mice (8 weeks old) were purchased from Changsha SLAC Laboratory Animal Company (Changsha, China, http://www.hnsja.com/) and were maintained on 12 h light/dark cycles with food and water available ad libitum at the Laboratory Animal Center of Nanchang University. All animal procedures described here were reviewed and approved by the Animal Care and Use Committee of Nanchang University.

For the purpose of cell tracking, hMBSCs and NIH 3T3 cells were labeled with PKH26 red fluorescent dye (Sigma, Aldrich) before coinjection with HeLa cells. The HeLa cells were then mixed with NIH 3T3 cells (control group) or hMBSCs (experimental group) at a 1 : 1 ratio (5 × 10^6^ : 5 × 10^6^ cells; *n* = 4) and injected subcutaneously into the dorsal region of BALB/c nude mice. Mice were anesthetized after 7 days, 14 days, and 21 days of cell injection and visualized with the whole-body fluorescent imaging system (LB983; Berthold, Germany). Mice were euthanized after 21 days of cell injection, and tumors were harvested and measured with a vernier caliper (Mitutoyo Co., Tokyo, Japan). The tumor volume was calculated using the following formula: (1/2)*ab*^2^ (*a*: longest size of tumor, *b*: shortest size of tumor).

### 2.11. Histopathology and TUNEL Assay

After 21 days of HeLa/NIH 3T3 or HeLa/hMBSC coinjection, mice were euthanized with pentobarbital, and tumor tissues were isolated and fixed in 4% paraformaldehyde, embedded in paraffin. Tissue was cut into 5 *μ*m-thick sections. After deparaffinization and rehydration, the sections were rinsed in PBS and then incubated in a 3% H_2_O_2_ solution to block the endogenous peroxidase. After incubation with 5% BSA for 30 min to block nonspecific antibody-binding sites, the samples were stained with PCNA primary antibody (1 : 1000, mouse monoclonal, Abcam) at 4°C overnight. The samples were rinsed with PBS twice and incubated with a HRP-conjugated goat anti-mouse secondary antibody (MaiXin biotechnologies, China) followed by visualization with 3,3-diaminobenzidine tetrahydrochloride (MaiXin biotechnologies). Finally, the sections were stained with hematoxylin and examined under a light microscope.

Apoptosis was analyzed on paraffinic tumor tissue sections of HeLa/NIH 3T3- and HeLa/hMBSC-coinjected groups by TUNEL assay kit (Millipore, USA). Three sections were selected for each mouse and stained using the TUNEL assay kit following the manufacturer's protocol. Under the microscope, cells with dark-brown nuclei were marked as positive and counted in 10 randomly selected fields per nude mouse with a total of 4 nude mice in each group.

### 2.12. Enzyme-Linked Immunosorbent Assay (ELISA)

The TGF-*β*1 expression in hMBSC-CM was performed using quantitative human ELISA kit (R&D Systems) following the manufacturer's protocol.

### 2.13. In Vitro Treatment with TGF-*β*1 Antibody and rhTGF-*β*1

TGF-*β*1 antibody and rhTGF-*β*1 were used to treat HeLa cells and to assess whether TGF-*β*1 contributed to cell cycle arrest. To neutralize TGF-*β*1, a TGF-*β*1-specific antibody was added into the transwell system at a final concentration of 10 ng/ml for 48 h. Nonspecific rabbit IgG was used as a negative control. rhTGF-*β*1 was used to treat HeLa cells for 48 h, and then cellular viability and cell cycle were analyzed.

### 2.14. Statistical Analysis

The results are presented as average value ± standard deviation (SD). Student's *t*-test was used for analysis between two groups. One-way analysis of variance (ANOVA) was used to compare data among three or more groups. Differences with a *P* value of < 0.05 were considered statistically significant.

## 3. Results

### 3.1. Morphology and Immunophenotyping of hMBSCs

**Figure 2:**
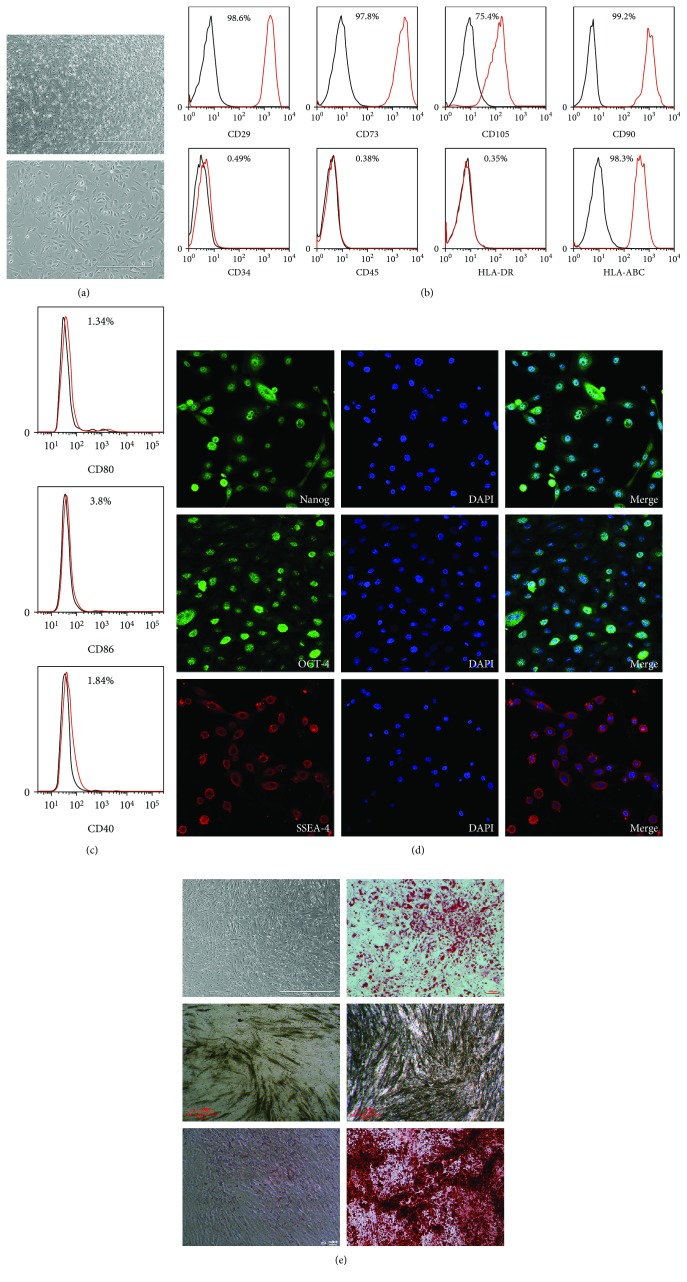
Characterization of cell morphology and markers of hMBSCs. (a) Phase-contrast microscopic images of cultured hMBSCs. (b) Detection of surface markers in hMBSCs (red) and in isotype controls (black) by flow cytometry. hMBSCs were positive for CD29, CD73, CD105, CD90, and HLA-ABC but negative for CD34, CD45, and HLA-DR. (c) The hMBSCs were negative for HLA-ABC costimulatory molecules CD80, CD86, and CD40. (d) Immunofluorescence staining showed almost all hMBSCs expressed the embryonic stem cell surface markers Oct4, SSEA-4, and Nanog. (e) Adipogenic differentiation of hMBSCs was demonstrated by staining with oil red O, and osteogenic differentiation was demonstrated by ALP staining at the middle stage and Alizarin Red staining at the late stage.

Cultured primary and passaged hMBSCs had a spindle-shaped, fibroblast-like morphology, and homogenous growth in monolayers. In the presence of bFGF (10 ng/ml), the hMBSCs proliferate robustly and the average doubling time was 2 days (Figure 2(a)). hMBSCs were positive for mesenchymal stem cell markers CD29, CD73, CD105, and CD90 and negative for hematopoietic stem cell markers CD34 and CD45 as determined by flow cytometry (Figure 2(b)). hMBSCs also expressed the major histocompatibility protein HLA-ABC but none of its costimulatory molecules CD80, CD86, and CD40 nor major histocompatibility protein HLA-DR (Figures 2(b) and 2(c)), indicating that these cells possess low immunogenicity. The expression of embryonic stem cell surface markers Nanog, Oct4, and SSEA-4 was also analyzed by immunofluorescence. Our results showed that hMBSCs express all of these pluripotent markers (Figure 2(d)), indicating hMBSCs have the capacity to self-renew as well as multilineage differentiation potentials. Under adipogenic and osteogenic differentiation conditions, hMBSCs were able to differentiate into adipocytes and osteocytes, respectively (Figure 2(e)).

### 3.2. hMBSCs Inhibit Proliferation, Migration, and Invasion of HeLa Cells In Vitro in a Paracrine Manner

In order to investigate the effect of hMBSCs and hMBSC-CM on the proliferation and invasion of HeLa cells in vitro, we compared the PBS control group, hMBSC-CM group, and hMBSC coculture group (Figure 1(a)). A cell count assay showed that hMBSC-CM (10%) and hMBSC coculture (at a ratio of HeLa cells : hMBSCs of 1 : 1) significantly decreased the cell number of HeLa cells at 48 h and 72 h (Figures 1(b) and 1(c)), indicating that hMBSC-secreted factors influenced the proliferation of HeLa cells. A CCK-8 assay further confirmed that a significant vitality inhibition in HeLa cells was induced by hMBSC-CM and hMBSC coculture compared to control at 48 h and 72 h (Figure 1(d)). To determine whether the effect of hMBSC-CM on the proliferation of HeLa cells was dose-dependent, HeLa cells were cultured with 3 ml H-DMEM complete medium supplemented with increasing amounts (2.5%, 5%, 10%, or 20%) of hMBSC-CM (10X) for 48 h. We found that HeLa cells exhibited a significant decrease in proliferation with 5%, 10%, and 20% hMBSC-CM (10X) (*P* < 0.05). In contrast, 2.5% hMBSC-CM (10X) did not significantly inhibit the growth of HeLa cells. When compared with the 10% hMBSC-CM treatment group, a slight but nonsignificant decrease in proliferation of HeLa cells was also observed in the 20% hMBSC-CM treatment group (Figure 1(e)). For the in vitro transwell coculturing experiments, HeLa cells were cultured in the presence of different concentrations of hMBSCs (at a ratio of HeLa cells : hMBSCs of 4 : 1, 2 : 1, 1 : 1, or 1 : 2). Proliferation was significantly reduced when the ratio of HeLa cells : hMBSCs was 1 : 1 and 1 : 2. However, no significant difference between the 1 : 1 group and the 1 : 2 group was found (Figure 1(f)). Thus, in subsequent experiments, we cultured HeLa cells in 10% CM, and in the transwell setting, HeLa cells were cultured at a 1 : 1 ratio with hMBSCs.

**Figure 3:**
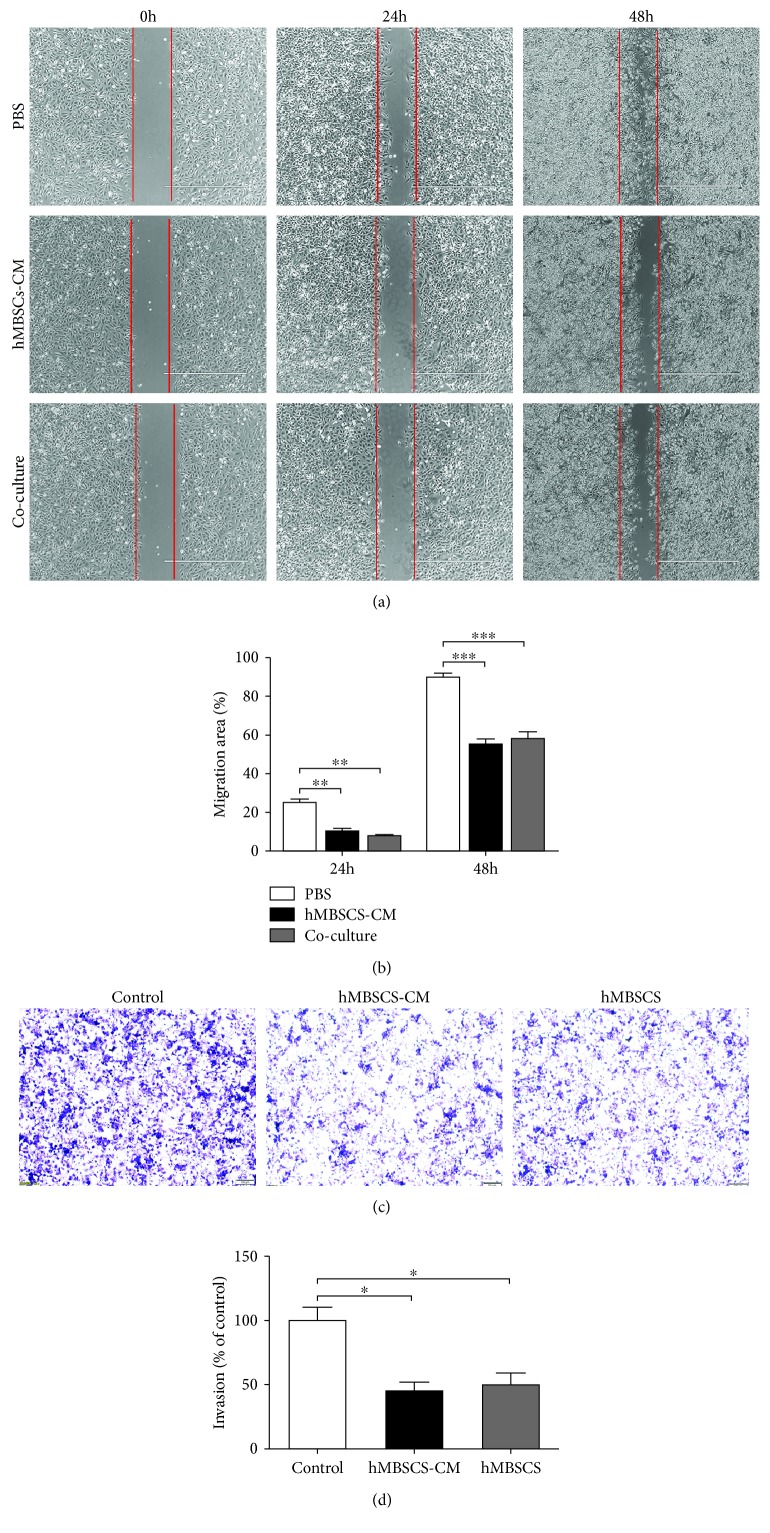
hMBSCs and hMBSC-CM inhibit the migration and invasion of HeLa cells in vitro in a paracrine manner. (a) Wound-healing assay for migration of HeLa cells in the PBS, hMBSC-CM, and hMBSC coculture groups. The red line indicates the initiatory areas without migrating cells. (b) Quantitative analysis of the migration area as shown in (a). (c) Matrigel invasion assay for invasion of HeLa cells in the control, hMBSC-CM, and hMBSC treatment group. The invaded HeLa cells were stained with crystal violet. (d) Quantitative analysis of the invaded cells as shown in (c). Significance was measured using a two-way ANOVA. ^∗^*P* < 0.05, ^∗^*P* < 0.01, ^∗∗∗^*P* < 0.001.

A scratch wound assay was used to determine the effect of hMBSC-CM and hMBSC coculture on the migration of HeLa cells. Compared to the PBS group, hMBSC-CM and hMBSC cocultures remarkably inhibited the migration of HeLa cells into the wound after 24 h and 48 h. However, no significant difference between the hMBSC-CM group and the hMBSC coculture group was observed (Figure 3(a)). At 24 h, the percent of wound closure was 10.5 ± 1.86% in the hMBSC-CM group and 8.1 ± 1.63% in the hMBSC coculture group, whereas in the PBS control group, it was 25.0 ± 2.14%. At 48 h, the percentages were 55 ± 4.08%, 58 ± 4.90%, and 90 ± 2.54%, respectively (Figure 3(b)). BD Matrigel™ invasion chambers were used for the invasion assay. The invasion of HeLa cells was significantly reduced in the 2-day coculture of hMBSC-CM and hMBSCs to 45 ± 6.3% and 50 ± 5.8% of control, respectively (Figures 3(c) and 3(d)). Collectively, these results demonstrate that hMBSCs can inhibit the proliferation, migration, and invasion of HeLa cells in vitro, in a paracrine manner.

### 3.3. hMBSCs Induced G0/G1 Cell Cycle Arrest in HeLa Cells In Vitro

**Figure 4:**
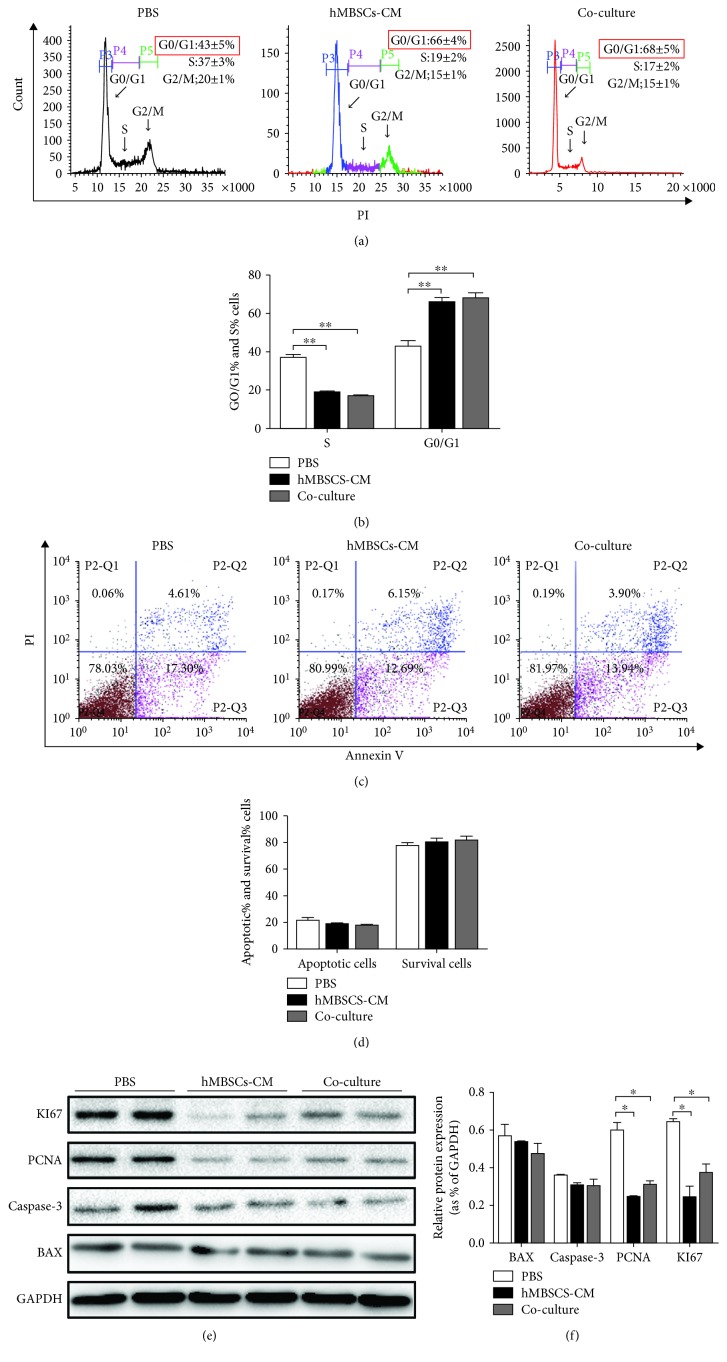
hMBSCs and hMBSC-CM induce cell cycle arrest of HeLa cells in vitro. (a) Cell cycle analysis through flow cytometry showed that hMBSC-CM and hMBSC coculture induces G0/G1 cell cycle arrest in HeLa cells. (b) Quantitative analysis of the percentage of cells in the G0/G1 and S phase of cell cycle as shown in (a) (*n* = 3). (c) HeLa cells were cultured under treatment of PBS, hMBSC-CM, and hMBSC coculture for 48 h and subjected to flow cytometry analysis for apoptotic cells (*n* = 3). (d) Quantitative analysis of the percentage of apoptotic cells and living cells as shown in (c) (*n* = 3). (e) Western blot for the KI67, PCNA, caspase-3, and Bax expression in HeLa cells after treatment with PBS, hMBSC-CM, and hMBSC coculture for 48 h. (f) Quantitative analyses for relative protein level of HeLa cells as shown in (e). Significance was measured using the two-way ANOVA. ^∗^*P* < 0.05, ^∗^*P* < 0.01, ^∗∗∗^*P* < 0.001.

To better understand the mechanisms involved in the antiproliferative activity of hMBSC-CM and hMBSC coculture, we performed apoptosis and cell cycle analyses. As shown in Figure 4(a), striking differences in cell cycle were observed, whereas most of the hMBSC-CM-treated and hMBSC cocultured HeLa cells had a significantly (*P* < 0.01) greater percentage of cells arrested in the G0/G1 phase (66 ± 4% and 68 ± 5%, respectively) compared to cells incubated with normal medium (43 ± 5%). Consistent with this, FACS analysis also revealed a lower proportion of HeLa cells in the S phase of the cell cycle in hMBSC-CM and hMBSC coculture groups (19 ± 2% and 17 ± 2%, respectively) compared to the PBS control group (37 ± 3%) (Figure 4(b)). In contrast, when compared with the PBS group, the rate of apoptotic cells was not significantly different in HeLa cells in response to hMBSC-CM treatment and hMBSC coculture (Figures 4(c) and 4(d)).

To confirm that hMBSC-secreted factors can induce cell cycle arrest in HeLa cells, we detected the expression of PCNA and KI67 (two proliferation-related proteins) by western blot analysis. The results show that the protein levels of PCNA and KI67 in HeLa cells were markedly decreased by hMBSC-CM and hMBSC coculture treatment, compared with that of the PBS-treated cells. The expression of two apoptosis-related proteins, caspase-3 and Bax, was not significantly different in hMBSC-CM and hMBSC coculture treatment HeLa cells (Figures 4(e) and 4(f)). These results indicate that the hMBSC-CM and hMBSC coculture can inhibit the proliferation of HeLa cells by inducing cell cycle arrest at the G0/G1 phase.

### 3.4. hMBSCs Inhibit Growth of HeLa Cells In Vivo

**Figure 5:**
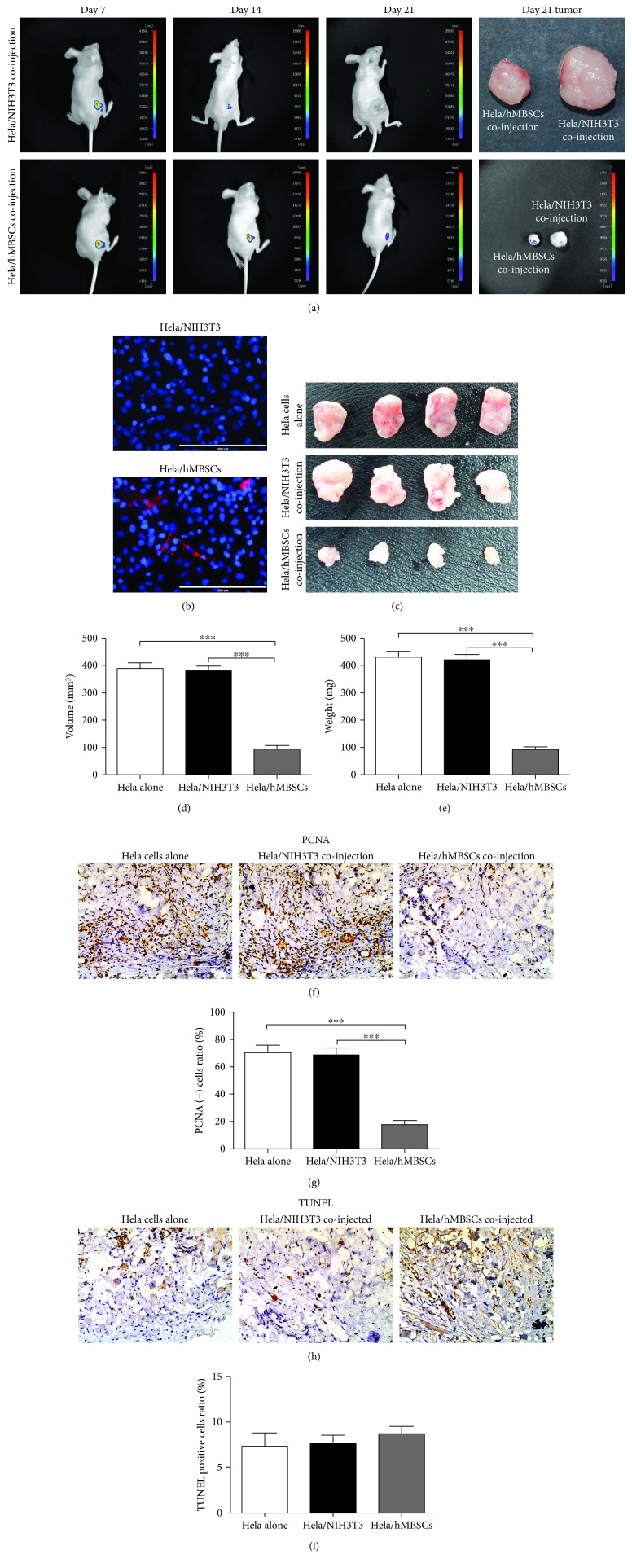
hMBSCs inhibit the growth of HeLa cells in vivo. (a) Whole-body fluorescent imaging analysis of PKH26 labeling in hMBSCs and NIH 3T3 cells in vivo. (b) The tumor tissues of the HeLa/NIH 3T3 and HeLa/hMBSC group were stained with DAPI and imaged by confocal microscopy. (c) Gross observation of subcutaneous xenografts of Hela cells alone, HeLa/NIH 3T3-coinjected, and HeLa/hMBSC-coinjected nude mice (*n* = 4). (d) The average volume of the HeLa/hMBSC-coinjected group was significantly smaller than that of the HeLa cells alone group and the HeLa/NIH 3T3-coinjected group at day 21 (*n* = 4). (e) The average weight of the HeLa/hMBSC-coinjected group was significantly lower than that of the HeLa cells alone group and the HeLa/NIH 3T3-coinjected group at day 21 (*n* = 4). (f) Proliferation of HeLa cells was tested by immunohistochemistry using antibodies against PCNA in HeLa cells alone, HeLa/NIH 3T3, and HeLa/hMBSC tumor tissues. (g) Quantification of PCNA-positive HeLa cells in (f). (h) Estimation of apoptosis in tumor tissues of the HeLa cells alone, HeLa/NIH 3T3-, and HeLa/hMBSC-coinjected groups using the TUNEL assay. (i) Quantification of TUNEL-positive HeLa cells in (h).^∗^*P* < 0.05, ^∗^*P* < 0.01, ^∗∗∗^*P* < 0.001.

The effects of hMBSCs and NIH 3T3 cells on HeLa cells in vivo were tested by subcutaneously coinjecting them with HeLa cells at a 1 : 1 ratio (5 × 10^6^ : 5 × 10^6^ cells; *n* = 5) into one side of the scapular region of a BALB/c nude mouse. HeLa cells alone were used as the control group. For cell tracking purposes, hMBSCs and NIH 3T3 cells were labeled with PKH26. To verify whether hMBSCs and NIH 3T3 cells were present in the tumor, mice were anesthetized after 7 days, 14 days, and 21 days of cell injection and visualized with a whole-body fluorescent imaging system. As shown in Figure 5(a), hMBSCs were clearly observed in the tumor tissue but were gradually reduced at 7, 14, and 21 days after injection. NIH 3T3 cells were also observed in the tumor at day 7 and day 14, but no NIH 3T3 cells were detected at day 21. Thus, at the time of sacrifice (day 21 after injection), there remained a few hMBSCs but no NIH 3T3 cells in the tumor. Mice were euthanized after 21 days of cell injection, and the tumor tissue was harvested. The immunostaining analysis showed that the PKH26-positive cells also expressed DAPI, suggesting that the hMBSCs present in the tumor were still alive (Figure 5(b)). We observed that hMBSCs significantly decreased the average volume and average weight of xenografted tumors compared with the HeLa cells alone group and the HeLa/NIH 3T3-coinjected group. In contrast, no significant difference between the HeLa cells alone group and the HeLa/NIH 3T3 group was observed (Figures 5(c)–5(e)). Results from an immunohistochemistry assay showed that hMBSCs significantly decreased the positive rates of PCNA in the HeLa/hMBSC-coinjected tumor tissues compared with those in the HeLa cells alone tissues and HeLa/NIH 3T3-coinjected tumor tissues (Figures 5(f) and 5(g)), which was consistent with the in vitro results. In order to test the treatment effect of hMBSCs on apoptosis in the tumor tissue, sections from the HeLa cells alone, HeLa/NIH 3T3-coinjected, and HeLa/hMBSC-coinjected groups were subjected to TUNEL staining. No significant reduction in cell apoptosis was observed in the HeLa/hMBSC group compared to the HeLa cells alone group and the HeLa/NIH 3T3 group, suggesting that hMBSCs do not accelerate the apoptosis of HeLa cells in vivo (Figures 5(h) and 5(i)). No significant difference in cell proliferation and apoptosis was observed in the HeLa/NIH 3T3 group compared to the Hela cells alone group (Figures 5(f)–5(i)).

### 3.5. The Suppressive Effect of hMBSCs on HeLa Cells Is Mediated through TGF-*β*1

**Figure 6:**
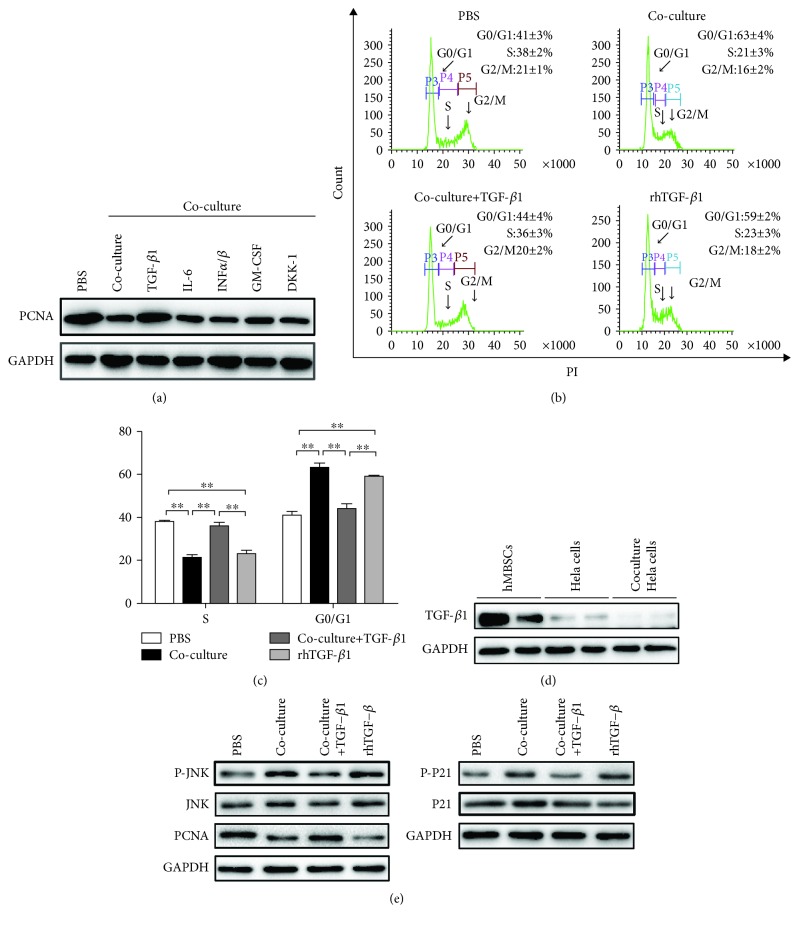
hMBSCs inhibit the growth of HeLa cells via TGF-*β*1-mediated signaling. (a) IL-6, INF*α*/*β*, GM-CSF, DKK-1, and TGF-*β*1 antibodies were added into the transwell system, and western blot was used to detect the expression of PCNA in HeLa cells for each treatment group. (b) Cell cycle analysis tested by flow cytometry showed that TGF-*β*1 antibody reversed hMBSC-induced cell cycle arrest in HeLa cells. (c) Quantitative analysis of the percentage of cells in the G0/G1 and S phase of cell cycle as shown in (b) (*n* = 3). (d) Western blot analysis of TGF-*β*1 protein levels in hMBSCs, HeLa cells, and HeLa cells cocultured with hMBSCs. (e) Western blot analysis of protein levels of PCNA, JNK, phospho-JNK, P21, and phospho-P21 in HeLa cells of each treatment group. ^∗^*P* < 0.05, ^∗^*P* < 0.01, ^∗∗∗^*P* < 0.001.

Interleukin-6 (IL-6) [38], *α*/*β* interferons (INF*α*/*β*) [39], granulocyte-macrophage colony-stimulating factor (GM-CSF) [40], dickkopf-1(DKK-1) [41], and TGF-*β*1 [25, 27, 42] are reported to be continuously secreted by MSCs and to participate in the mechanisms involved in the control of cell proliferation. To determine which hMBSC-derived factor contribute to the cell cycle arrest of HeLa cells, we added IL-6, INF*α*/*β*, GM-CSF, DKK-1, and TGF-*β*1 antibody into the transwell system. We found that the inhibitory effect of hMBSC coculture could only be suppressed by TGF-*β*1 antibody, indicating that hMBSC-secreted TGF-*β*1 plays an important role in the induction of cell cycle arrest in HeLa cells (Figure 6(a)). To confirm whether the cell cycle arrest of HeLa cells induced by hMBSCs was mediated by TGF-*β*1, we first detected the TGF-*β*1 concentration in hMBSC-CM by ELISA. Our results showed that a substantial amount of TGF-*β*1 (1057 pg/10^5^ cells, 5.29 ng/ml) was secreted into the culture medium by hMBSCs within 48 h. The medium without FBS was set as a negative control. Furthermore, we added TGF-*β*1 antibody (10 ng/ml) into the transwell system and evaluated the cell cycle progression of HeLa cells at 48 h. Our results showed that TGF-*β*1 antibody significantly decreased the proportion of HeLa cells in the G0/G1 phase from 63 ± 4% (coculture group) to 44 ± 4% (coculture+TGF-*β*1 group) (*P* < 0.01). A significant increase (*P* < 0.01) from 21 ± 3% to 36 ± 3% of the S phase cells was also observed when TGF-*β*1 antibody was added into the transwell system. These results indicate that TGF-*β*1 antibody reversed hMBSC-induced cell cycle arrest. To further confirm the effects of TGF-*β*1 on cell cycle progression in HeLa cells, rhTGF-*β*1 was added to treat HeLa cells and cell cycle was evaluated by flow cytometry. When compared with the PBS group, rhTGF-*β*1 induced a significant decrease in the S phase and a significant increase in the G0/G1 phase in HeLa cells (Figures 6(b) and 6(c)). To confirm that TGF-*β*1 in the coculture system was secreted by hMBSCs and not HeLa cells, western blot analysis was used to detect the expression of TGF-*β*1 in hMBSCs, HeLa cells, and HeLa cells cocultured with hMBSCs. We found that hMBSCs had high expression of TGF-*β*1. In contrast, both conditions of HeLa cells were negative for TGF-*β*1 (Figure 6(d)).

Phosphorylation of JNKs is associated with protein stabilization of P21 [43], which is a negative regulator of the cell cycle and might be associated with TGF-*β*1-induced growth inhibition [44]. Western blot analysis showed that hMBSCs significantly decreased the expression of PCNA and increased the expression of phospho-JNK and phospho-P21 in cocultured HeLa cells compared with the PBS control group. In HeLa cells treated with hMBSCs+TGF-*β*1 antibody, a reduced level of phospho-JNK and phospho-P21 was observed compared to HeLa cells only treated with hMBSCs. The expression level of phospho-JNK and phospho-P21 was also upregulated in the rhTGF-*β*1-treated cells (Figure 6(e)). These results indicate that the cervical carcinoma suppressive effect derived from hMBSCs is mediated by TGF-*β*1 and subsequent upregulation of phospho-JNK and phospho-P21 signaling cascades.

## 4. Discussion

hMBSCs are a newly identified type of MSCs and have many important advantages such as a noninvasive isolation procedure, low immunogenicity, no oncogenicity, high proliferative potential, and no ethical conflicts. In this study, we found that hMBSCs expressed high levels of three core pluripotency proteins (OCT4, SSEA-4, and Nanog) and the MSC markers CD29, CD73, CD105, and CD29. However, hMBSCs do not express the hematopoiesis-specific markers CD34 and CD45 (Figure 2(b)). Under adipogenic and osteogenic differentiation conditions, the hMBSCs had the potential to differentiate into adipocytes and osteocytes, respectively (Figure 2(e)). These observations indicate that hMBSCs have similar characteristics to BM-MSCs and have multilineage differentiation potential. The human leukocyte antigen (HLA) system represents the loci of genes that play a crucial role in determining donor-recipient immune compatibility in organ transplantation [45]. Very low levels of HLA class I (HLA-A/B/C) and II (HLA-DR and DQ) molecules have been reported in hMBSCs [28]. Our results have also shown that the hMBSCs were negative for HLA-DR, CD80, CD86, and CD40 and have low expression of HLA-ABC (Figures 2(b) and 2(e)), suggesting weak immunogenicity and potential immune tolerance after transplantation of hMBSCs. These characteristics make hMBSCs ideal candidates for cancer therapy.

There have been reports showing that human endometrial mesenchymal stem cells derived from menstrual blood attenuate epithelial ovarian cancer (EOC) growth by inducing cell cycle arrest and promoting apoptosis in EOC cells in vitro and in vivo [46]. Whether hMBSCs provide antitumor properties to other cancer cells, especially cervical cancer, has never been reported. In this paper, we test the antitumor properties of hMBSCs on cervical cancer cells in vitro and in vivo. In recent reports, CM derived from adipose MSCs [47] and human ESCs [48] was found to inhibit proliferation but not cell death in hepatocellular carcinoma (HCC), ovarian cancer, and prostate cancer. To elucidate whether hMBSCs inhibit tumor proliferation through paracrine signaling, hMBSC was used in a transwell coculture system to detect the effect of hMBSC-secreted factors on the proliferation of HeLa cells. First, cells were either treated with PBS or cocultured with NIH 3T3. When compared with the PBS group, we found that the NIH 3T3 coculture did not affect the proliferation, migration, and invasion of HeLa cells. Therefore, in order to reduce the number of experimental groups, we only used the PBS group as the negative control for in vitro experiments. In agreement with previous reports, we found that both hMBSC-CM and hMBSC coculture could inhibit the proliferation, migration, and invasion of HeLa cells in vitro (Figures 1 and 3). However, we did not observe a significant level of cell death mediated by hMBSCs (Figures 4(c)–4(f)). We also found that hMBSCs decreased the expression of PCNA and KI67 in HeLa cells treated with hMBSC-CM or cocultured with hMBSCs. Furthermore, flow cytometric analysis showed that hMBSC-CM and hMBSC coculture significantly induced G0/G1 cell cycle arrest in HeLa cells with a significant decrease in cells in the S phase. There was no significant difference observed in HeLa cells treated with hMBSC-CM or cocultured with hMBSC. We also demonstrated the ability of hMBSCs to inhibit HeLa cell proliferation in vivo.

We coinjected HeLa cells with NIH 3T3 cells or hMBSCs into BALB/c nude at a 1 : 1 ratio, and HeLa cells alone were used as control. Whole-body fluorescent imaging analysis showed that hMBSCs were present in the tumor tissue and were gradually reduced at 7, 14, and 21 days after injection. NIH 3T3 cells were also observed in the tumor at day 7 and day 14, but no NIH 3T3 cells were detected at day 21 (Figure 5(a)). The average volume and average weight of tumors decreased in the HeLa/hMBSC group compared to the HeLa cells alone group and the HeLa/NIH 3T3 group (Figures 5(c)–5(e)). These data suggest that the inhibitory factors secreted from hMBSCs play an important role in the growth inhibition of HeLa cells [27].

The specific soluble antiproliferative factors secreted by MSCs remain unknown. TGF-*β*1 is well recognized as a potent inhibitor of cell proliferation of endothelial, epithelial, and cancer cells [49]. Chen et al. found that TGF-*β*1 inhibited cell growth and DNA synthesis and induced G0/G1 cell cycle arrest [50]. Bu et al. found that human amniotic epithelial cells secreted abundant TGF-*β*1, decreased the proliferation of epithelial ovarian cancer cells, and induced G0/G1 cell cycle arrest in cancer cells in vivo and in vitro [27]. IL-6 [38], INF*α*/*β* [39], GM-CSF [40], and DKK-1 [41] are reported to be continuously secreted by MSCs and to participate in the mechanisms involved in the control of cell proliferation. To determine which hMBSC-derived factors contribute to the cell cycle arrest of HeLa cells, we added TGF-*β*1, IL-6, INF*α*/*β*, GM-CSF, and DKK-1 antibodies into the transwell system. We found that the inhibitory effect of the hMBSC coculture could only be suppressed by the TGF-*β*1 antibody (Figure 6(a)), indicating that hMBSC-secreted TGF-*β*1 plays an important role in the induction of cell cycle arrest in HeLa cells. Next, we assessed the changes in proliferation in response to treatment of HeLa cells with rhTGF-*β*1. Results showed that rhTGF-*β*1 induced a significant decrease in the S phase and a significant increase in the G0/G1 phase of HeLa cells (Figures 6(b) and 6(c)). The results confirmed our hypothesis that TGF-*β*1 secreted by hMBSCs was responsible for the inhibition of HeLa cell proliferation potential.

In some instances, TGF-*β*1 has been reported to regulate phosphorylation of JNKs, which coordinate cell responses to stress and influence regulation of cell growth and transformation [27]. Umbilical cord tissue-derived MSCs induce apoptosis in PC-3 prostate cancer cells through activation of JNK and downregulation of PI3K/AKT signaling [51]. Although we found that the suppressive effect of hMBSC-CM and hMBSC coculture in HeLa cells is exerted through an inhibition on cell proliferation and not by regulating apoptosis, the expression level of phospho-JNK in the hMBSC-CM and hMBSC coculture group was significantly increased compared to control. Nevertheless, findings from our study have shown that the expression levels of PI3K and AKT were not significantly different between these three groups. P21, stabilized by JNK [52], is a critical regulator of tumorigenesis and suppresses tumors by regulating cell cycle arrest and/or apoptosis [53]. Magatti et al. found that the expression of P21 can be upregulated by MSCs and subsequent inhibition of cyclins and cyclin-dependent kinases (CDKs), leading to cell cycle arrest [25]. Our data demonstrated that the activation of phospho-JNK upregulated the expression of phospho-P21 in hMBSC-CM and rhTGF-*β*1-treated HeLa cells (Figure 6(e)). These results indicate that hMBSCs inhibit the proliferation of HeLa cells via TGF-*β*1-mediated JNK/P21 signaling.

## 5. Conclusion

We have found novel intrinsic anticervical cancer properties of hMBSCs in vivo and in vitro. Furthermore, we showed that hMBSCs secrete high levels of TGF-*β*1, which sequester and inhibit HeLa cell proliferation by inducing cell cycle arrest. The inhibitory effect of TGF-*β*1 is evident from the increased level of phospho-JNK and phospho-P21 and from reduced HeLa cell proliferation. This study supports the use of hMBSC-based antitumor therapy against cervical cancer.

## Data Availability

The data used to support the findings of this study are included within the article. Any additional information about the data used to support the findings of this study are available from the corresponding author upon request.
